# Toward Efficient Beige Adipogenesis: Protocol Optimization Using Adipose-Derived Stem Cells

**DOI:** 10.3390/cells15010054

**Published:** 2025-12-28

**Authors:** Klaudia Simka-Lampa, Agnieszka Kosowska, Wojciech Garczorz, Małgorzata Kimsa-Furdzik, Grzegorz Wystrychowski, Celina Kruszniewska-Rajs, Małgorzata Muc-Wierzgoń, Tomasz Francuz

**Affiliations:** 1 Department of Biochemistry, Faculty of Medical Sciences in Katowice, Medical University of Silesia, Medyków 18, 40-752 Katowice, Poland; 2 Department of Nephrology, 4th Provincial Hospital, 41-902 Bytom, Poland; 3 Department of Molecular Biology, Faculty of Pharmaceutical Sciences in Sosnowiec, Medical University of Silesia, Jedności 8, 41-200 Sosnowiec, Poland; 4 Department of Internal Diseases Propaedeutics and Emergency Medicine, Faculty of Public Health in Bytom, Medical University of Silesia, Piekarska 18, 44-902 Bytom, Poland

**Keywords:** adipogenesis, beige adipocytes, brown adipose tissue, adipose-derived stem cells, differentiation, uncoupling protein 1 (UCP1), thermogenin

## Abstract

Brown adipose tissue (BAT) has emerged as a promising therapeutic target for metabolic disorders such as type 2 diabetes and obesity. To advance research on BAT activation and elucidate the mechanisms underlying adipogenesis, it is crucial to develop a reliable in vitro model. This study aimed to optimize the differentiation of adipose-derived stem cells (ADSCs) into beige adipocytes and to validate the protocol using primary human ADSCs obtained from eight donors. Protocol optimization was first performed with commercial ADSCs, testing more than 30 combinations of adipogenic conditions. Differentiation was assessed by microscopy, Oil Red O staining, and uncoupling protein 1 (UCP1) expression via reverse transcription-quantitative polymerase chain reaction (RT-qPCR) and Western blot. Among the key adipogenic factors, rosiglitazone proved more effective than indomethacin. Extending the induction phase from 4 to 8 days and maintaining dexamethasone throughout the culture markedly enhanced differentiation efficiency. Serum concentration above 5% was inhibitory, while optimal conditions were identified as 5 μM rosiglitazone and 20 μg/mL insulin. The optimized protocol successfully induced beige adipogenesis in ADSCs from eight independent donors, though efficiency varied considerably which could be attributed to individual donor variability. These findings provide a robust in vitro model for studying beige fat biology and highlight the relevance of personalized approaches in metabolic research.

## 1. Introduction

Brown adipose tissue (BAT) plays a pivotal role in thermoregulation in newborns and young children by mediating non-shivering adaptive thermogenesis through the expression of uncoupling protein 1 (UCP1, thermogenin), which is responsible for uncoupling oxidative phosphorylation in mitochondria. BAT decreases with age and gradually transforms into white adipose tissue (WAT) [1]. Due to advances in modern research methods, BAT has been confirmed to persist in various adult tissues [2,3] and shown to play a key role in metabolic regulation [3,4], overturning the earlier belief that it regressed completely with age. Studies in animal models have shown that BAT transplantation restores euglycemia, lowers levels of pro-inflammatory cytokines such as interleukin-6 (IL-6) and tumor necrosis factor-alpha (TNF-α), and reverses clinical symptoms of diabetes [5]. Moreover, it reduces body weight by increasing energy expenditure without altering food intake [6] and attenuates the detrimental effects induced by a high-fat diet [7].

BAT has been recognized as a promising therapeutic target for metabolic disorders, including obesity, insulin resistance, and diabetes [8]. Moreover, it has been demonstrated that BAT exerts beneficial effects on cardiometabolic health [9] and may help alleviate symptoms associated with polycystic ovary syndrome (PCOS) [10]. Consequently, intensive research efforts are focused on therapeutic approaches, such as in vivo activation of BAT, induction of white adipose tissue browning [11], and cell-based therapies employing BAT progenitors [12]. These developments highlight the critical need to further elucidate the mechanisms governing BAT activation, alongside the establishment of optimal experimental models for studying adipogenesis and beige adipocyte metabolism.

This study focuses on the potential of adipose-derived stem cells (ADSCs) to differentiate into beige adipocytes. The latter cells exhibit key characteristics of brown adipocytes, yet differ in their origin [13]. A notable advantage of ADSCs is their high self-renewal capacity and ability to differentiate into various cell lineages. They can be obtained through a minimally invasive procedure from adipose tissue, providing a significantly higher yield of cells compared to bone marrow-derived mesenchymal stem cells (BM-MSCs) [14]. In contrast to embryonic stem cells, collection of ADSCs does not raise ethical concerns, and the ability to use them in autologous therapies further reduces the risk of transplant rejection.

To date, no single, universal, and fully optimized protocol for differentiating ADSCs or other mesenchymal stem cells (MSCs) into BAT cells has been established that could be regarded as a “gold standard”. Current differentiation protocols primarily rely on the use of rosiglitazone or indomethacin as the key differentiating agents. However, these protocols vary considerably in media composition, concentrations of differentiating factors, and culture duration (Supplementary Table S1) [15,16,17,18,19,20,21,22,23,24,25,26,27,28,29,30]. The majority of studies are conducted using commercial cells or primary cells derived from animals. Research involving primary human ADSCs remains limited.

In this study, we optimized the differentiation protocol to reliably derive beige adipocytes from ADSCs, building on established methodologies, with the aim of improving efficiency, reproducibility, and standardization, while enabling scalable applications for downstream in vitro analyses. A distinctive aspect of our work is the application of primary human ADSCs to evaluate the efficacy of the optimized differentiation strategy. Considering the heterogeneity of ADSCs and the variability in cellular responses to environmental stimuli, the validation of the protocol was performed using cells obtained from eight independent donors.

## 2. Materials and Methods

### 2.1. Cell Culture

#### 2.1.1. Differentiation of Commercial ADSCs Towards Beige Adipocytes

**Figure 1:**
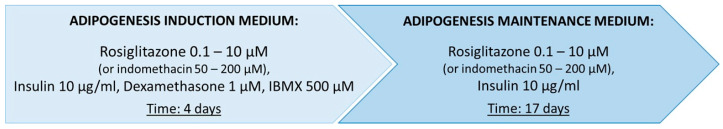
Baseline protocol for the differentiation of mesenchymal stem cells (MSCs) into brown adipocytes, applying either rosiglitazone or indomethacin, selected for optimization based on literature data (Supplementary Table S1) [15,16,17,18,19,20,21,22,23,24,25,26,27,28,29,30]. Used abbreviations—3-isobutyl-1-methylxanthine (IBMX).

A commercial human ADSC (Lonza, Basel, Switzerland) at passage 4 was seeded into 6-well plates at a density of 100,000 cells per well. The cells were cultured in Dulbecco’s Modified Eagle Medium (DMEM) (Lonza, Basel, Switzerland) supplemented with 10% fetal bovine serum (FBS) and 1% antibiotic solution containing penicillin (10,000 U/mL), streptomycin (10 mg/mL), and amphotericin B (25 µg/mL) under standard conditions: at 37 °C in a humidified atmosphere with 5% CO_2_ until full confluency was reached. Two days after reaching confluency (T0), ADSC differentiation into beige adipocytes was initiated following a literature-based protocol (Figure 1). Cells were cultured in DMEM/F-12 supplemented with 5% FBS, 1% antibiotics, and differentiation factors: rosiglitazone (or indomethacin), 3-isobutyl-1-methylxanthine (IBMX), insulin, and dexamethasone. The medium was refreshed every 2–3 days, and cell morphology was monitored using a ZOE Fluorescent Cell Imager (Bio-Rad, Hercules, CA, USA). Unless otherwise specified, all reagents were purchased from Merck (Darmstadt, Germany).

**Figure 2:**
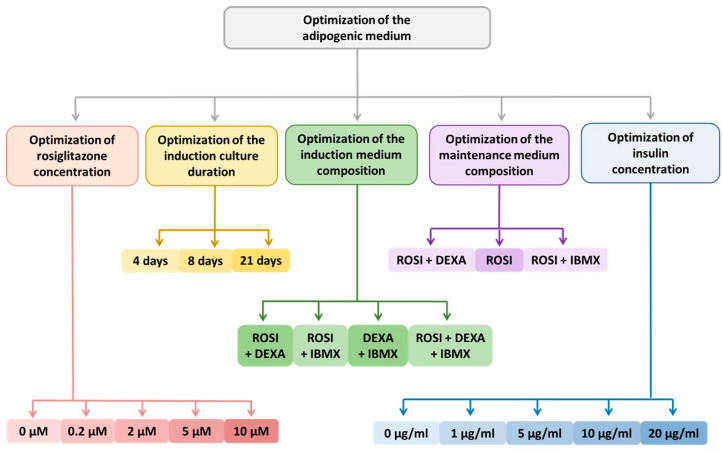
Schematic representation of the optimized protocol for the differentiation of adipose-derived stem cells (ADSCs) into beige adipocytes, driven by rosiglitazone as the key differentiation factor. Used abbreviations—dexamethasone (DEXA), 3-isobutyl-1-methylxanthine (IBMX), rosiglitazone (ROSI).

The baseline differentiation protocol was subjected to numerous modifications (Supplementary Table S2). Optimization of the differentiation process toward beige adipocytes was performed using rosiglitazone (Figure 2) and indomethacin. Additionally, the study evaluated the spontaneous differentiation capacity of ADSCs into adipocytes and the individual effects of specific differentiation factors on ADSC morphology.

Differentiation cultures for each medium variant were conducted in multiple replicates—at least three technical and biological repeats—at different time points to validate the obtained results. In some cases, cultures using different medium variants were carried out simultaneously to enable real-time comparison of differentiation effects throughout the experiment. Two experimental control groups were included: ADSCs cultured until T0—two days after reaching full confluency (K0), and ADSCs cultured for the entire duration of the experiment—21 days post T0 (K-21D). The control groups were cultured in DMEM/F-12 medium supplemented with 5% FBS and 1% antibiotic solution, under standard conditions, without the addition of differentiation factors.

Only the most effective differentiation conditions—those resulting in the highest number of lipid droplets stained with Oil Red O, indicative of beige phenotype—were selected for subsequent protein quantification and reverse transcription-quantitative polymerase chain reaction (RT-qPCR) analysis.

#### 2.1.2. Isolation and Differentiation Culture of Primary ADSCs

Subcutaneous adipose tissue was collected from eight adult donors of both sexes during liposuction or intraoperatively as part of planned surgical procedures. All donors were at least 30 years old and free of diseases of carbohydrate metabolism. Written informed consent was obtained from all participants prior to inclusion. Donor characteristics are presented in Table 1. Exclusion criteria were acute inflammatory conditions, history of psychiatric illness, chronic use of medications (including anti-inflammatory or analgesic drugs and proton pump inhibitors), had diagnosed cancer, chronic nicotine/alcohol use, or use of addictive substances. The study protocol was approved by the Bioethical Committee of the Medical University of Silesia.

Mesenchymal stem cells were isolated from adipose tissue through mechanical homogenization combined with enzymatic digestion using collagenase type I. Briefly, adipose tissue samples were repeatedly washed with an antibiotic solution, finely minced with a scalpel, and digested in 0.1% collagenase type I (Sigma-Aldrich, St. Louis, MO, USA) prepared in DPBS containing 1% bovine serum albumin (BSA) at 39 °C for 1–2 h under constant agitation (200 rpm). Enzymatic activity was neutralized by adding an equal volume of DMEM supplemented with 10% FBS. The digested suspension was centrifuged at 1200 rpm for 5 min at room temperature, and the stromal vascular fraction (SVF) containing ADSCs was collected. The SVF pellet was resuspended in DMEM supplemented with 10% FBS and 1% antibiotics and cultured under standard conditions (see Section 2.1.1). Non-adherent cells and tissue debris were removed by washing with DPBS during medium changes. Upon reaching confluency, adherent ADSCs were passaged and cryopreserved after the first passage in freezing medium consisting of DMEM supplemented with 10% FBS and 10% dimethyl sulfoxide (DMSO). After thawing, the cells were cultured under standard conditions, and subsequently seeded into 6-well plates at a density of 100,000 cells per well. At time point T0, differentiation was initiated using the optimized protocol: an 8-day culture in induction medium: DMEM/F-12 (Lonza, Basel, Switzerland) supplemented with 5% FBS, 1% antibiotic solution, 1 μM dexamethasone, 500 μM IBMX, 5 μM rosiglitazone, and 20 μg/mL insulin, followed by a 13-day culture in maintenance medium of the same composition, excluding IBMX.

#### 2.1.3. Oil Red O Staining of Lipid Droplets

Oil Red O specifically binds to the lipids found in lipid droplets, which are hallmark structures of mature adipocytes. After the differentiation culture was completed, the cells were fixed with 4% formaldehyde solution in physiological saline (0.9% NaCl). Subsequently, the cells were stained with a 0.2% Oil Red O solution in 60% isopropanol. After rinsing off the unbound dye, the cells were analyzed microscopically and photographic documentation was performed.

### 2.2. Image-Based Quantification of Differentiated Cells

Image analysis was conducted using ImageJ 1.54g, an open-source image processing software written in Java 8 and released into the public domain (developed and distributed by the National Institutes of Health, Bethesda, MD, USA), to quantify the percentage of differentiated cells. For each experimental condition (or each donor-derived ADSC sample), five randomly selected microscopic fields were analyzed. Differentiation efficiency was determined by calculating the proportion of the total image area occupied by cells exhibiting characteristic lipid droplet accumulation.

### 2.3. Flow Cytometry Analysis

Verification of the commercial ADSCs as well as primary ADSCs obtained from patients was performed based on the presence of characteristic surface markers using the Human Mesenchymal Stem Cell Multi-Color Flow Cytometry Kit (R&D Systems, Minneapolis, MI, USA). Flow cytometric analysis was conducted on the Attune NxT (Thermo Fisher Scientific, Waltham, MA, USA) for the commercial cells and on the FACSCanto II flow cytometer (Becton Dickinson Biosciences, Franklin Lakes, NJ, USA) for primary ADSCs as described in Wystrychowski et al., 2024 [31].

### 2.4. Analysis of UCP1 Gene Expression by RT-qPCR

Total RNA extraction was performed using TRIzol reagent (Invitrogen Life Technologies, Carlsbad, CA, USA), following the manufacturer’s protocol. Quantitative and qualitative assessment of the obtained RNA extracts was conducted using an Infinite M200 Pro spectrophotometer (Tecan, Männedorf, Switzerland).

Expression analysis of the UCP1 gene in the studied groups was carried out using RT-qPCR with a Light Cycler 480 sequence detector (Roche, Basel, Switzerland). As endogenous controls the gene encoding glyceraldehyde-3-phosphate dehydrogenase (GAPDH) were used. The GoTaq Probe 1-Step RT-qPCR System (Promega, Madison, WI, USA) was utilized to perform the reaction. TaqMan molecular probe sets labeled with 6-carboxyfluorescein (FAM) and primers complementary to the analyzed genes were used: UCP1 (ID: Hs01084772_m1) and GAPDH (ID: Hs99999905_m1) (Thermo Fisher Scientific, Waltham, MA, USA). Each sample was analyzed in two technical replicates, and the average value was used for analysis. Gene expression levels of UCP1 were determined using the relative 2^−ΔΔCT method. RT-qPCR analysis of UCP1 gene expression was performed on selected ADSC samples before and after differentiation toward beige adipocytes.

### 2.5. Semi-Quantitative Assessment of UCP1 Protein by Western Blot

Cell lysis was performed using RIPA buffer supplemented with protease inhibitors (Merck KGaA, Darmstadt, Germany). The total protein concentration in the cell lysate was assessed using the BCA method and measured with the Infinite M200 Pro microplate reader (Tecan, Männedorf, Switzerland).

Electrophoretic separation of proteins was performed by SDS-PAGE using a discontinuous two-layer polyacrylamide gel, consisting of a 4% stacking gel and a 12% resolving gel, followed by electrotransfer onto a PVDF membrane (Millipore, Burlington, MA, USA). The membrane was incubated overnight at 4 °C with mouse anti-UCP1 antibody (R&D Systems, Minneapolis, MI, USA) at a concentration of 0.5 µg/mL and rabbit anti-GAPDH antibody (Merck KGaA, Darmstadt, Germany) at a concentration of 0.1 μg/mL. Semi-quantitative analysis of the samples was performed based on the measurement of fluorescence intensity of the fluorochrome conjugated to the secondary antibodies: anti-mouse and anti-rabbit (Li-Cor) using the LiCor Odyssey Imaging System (Li-Cor Biosciences, Lincoln, NE, USA). Antibodies were diluted in Odyssey Blocking Buffer (Li-Cor Biosciences, Lincoln, NE, USA).

### 2.6. Statistical Analysis

The statistical analysis was conducted using Statistica software 13.3 (StatSoft, Tulsa, OK, USA). The normality of data distribution was assessed with the Shapiro–Wilk test, and homogeneity of variances was verified using Levene’s test. For RT-qPCR data, one-way analysis of variance (ANOVA) was used to evaluate differences in gene expression between the studied groups, followed by the post- hoc Tukey’s test. To determine statistically significant differences in the dependent variables before and after differentiation, a paired-samples *t*-test was applied. Statistical significance was defined as *p* < 0.05. Quantitative analysis of microscopic images (percentage of the area occupied by differentiated cells) was also performed using one-way ANOVA with Tukey’s post hoc test.

## 3. Results

### 3.1. Flow Cytometry Analysis of the Commercial and Primary ADSCs

**Figure 3:**
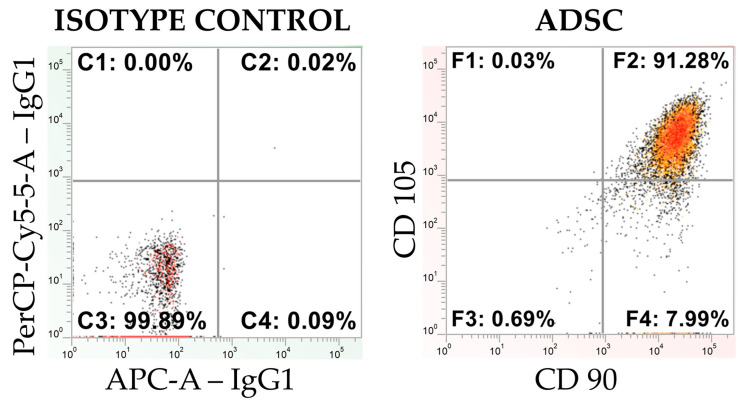
A cytometric dot plot illustrating the isotype control and the expression of MSC markers CD90 and CD105 for the CD45-negative population in an ADSC line (obtained from Lonza), presented on a logarithmic scale.

Flow cytometry analysis revealed that cells from the commercial ADSC line at passage 4 exhibited strong expression of the surface markers CD90 and CD105. In contrast, no expression of the surface protein CD45 or the protein CD146 was detected (Figure 3). These findings confirm the mesenchymal stem cell identity.

Similarly, flow cytometric analysis of human-derived ADSCs confirmed their mesenchymal stem cell identity, demonstrating a marker expression profile consistent with that observed in the commercial cells. The detailed characterization of these primary ADSCs has been comprehensively reported by Wystrychowski et al. [31].

### 3.2. Comparative Morphological Assessment of ADSCs Differentiated into Beige Adipocytes Using Different Protocols

No spontaneous differentiation of ADSCs was observed during prolonged culture (21 days post-T0). Culturing ADSCs in the presence of only one of the following differentiation factors: 10 µg/mL insulin, 1 μM dexamethasone, 500 µM IBMX, 0.2 µM rosiglitazone, or 100 μM indomethacin did not result in the formation of lipid droplets (Supplementary Figure S1).

#### 3.2.1. Optimization of the Differentiation Culture Protocol of ADSCs Towards Beige Adipocytes with Rosiglitazone

##### Optimization of the Induction Medium Composition

Culture of ADSCs for 21 days in the presence of dexamethasone and IBMX resulted in a morphological change; however, adipogenesis did not occur, as evidenced by the lack of lipid droplets within the cells. The application of both rosiglitazone and IBMX or rosiglitazone and dexamethasone led to the differentiation of individual cells into adipocytes, as indicated by the presence of small lipid droplets. Nevertheless, the highest efficiency in terms of the number of differentiated cells and the amount of lipid droplets within them was achieved when the initial differentiation protocol was applied (containing IBMX, dexamethasone, and rosiglitazone in the induction medium) (Supplementary Figure S2).

##### Optimization of the Induction Culture Duration

**Figure 4:**
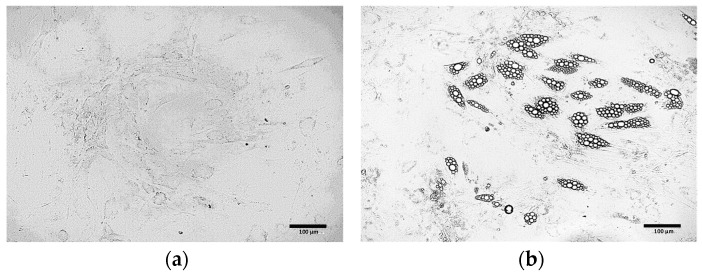

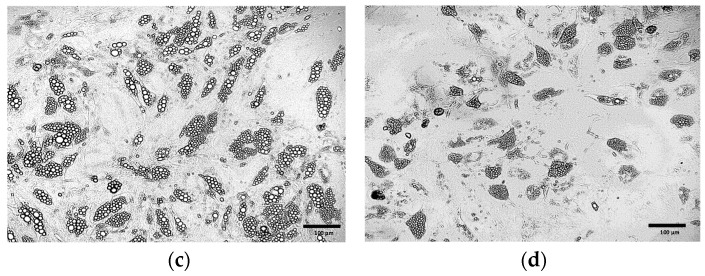
ADSCs cultured in induction medium for the following periods of time: (**a**) 0 days (21 days in maintenance medium only); (**b**) 4 days; (**c**) 8 days; (**d**) 21 days.

Extending the duration of the induction culture from 4 (Figure 4b) days to 8 days (Figure 4c) resulted in the increased number of cells exhibiting the phenotype of beige adipocytes. Nevertheless, the use of the induction medium throughout the entire culture period (21 days post T0) initially led to the formation of numerous lipid droplets and changes in cell morphology (Figure 4d). However, over time, a gradual cell death was observed. Exclusion of the induction medium prevented the differentiation of ADSCs into adipocytes (Figure 4a).

##### Optimization of Insulin Concentration

Morphological analysis of the cells after completion of the differentiation culture demonstrated that the optimal insulin concentration in the adipogenic medium was 10 μg/mL (Supplementary Figure S3).

##### Optimization of the Maintenance Medium Composition

**Figure 5:**
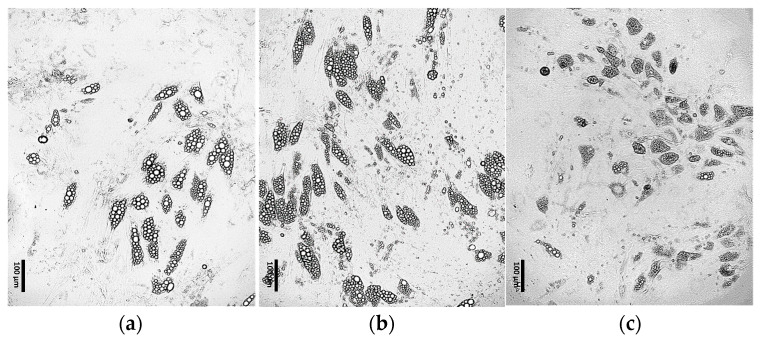
ADSCs cultured for 4 days in induction medium, followed by 17 days in differentiation medium supplemented with: (**a**) 0.2 μM rosiglitazone and 10 μg/mL insulin; (**b**) 0.2 μM rosiglitazone, 10 μg/mL insulin and 1 μM dexamethasone; (**c**) 0.2 μM rosiglitazone, 10 μg/mL insulin and 500 μM IBMX.

It was observed that the continuous addition of 1 μM dexamethasone to the differentiation medium throughout the entire culture enhanced the efficiency of ADSC differentiation into adipocytes (Figure 5b) compared to the initial differentiation protocol (Figure 5a). In contrast, the continuous addition of 500 μM IBMX initially promoted increased differentiation efficiency and lipid droplet formation; however, over time, it led to marked changes in cell morphology followed by progressive cell death (Figure 5c).

##### Optimization of Rosiglitazone Concentration

**Figure 6:**
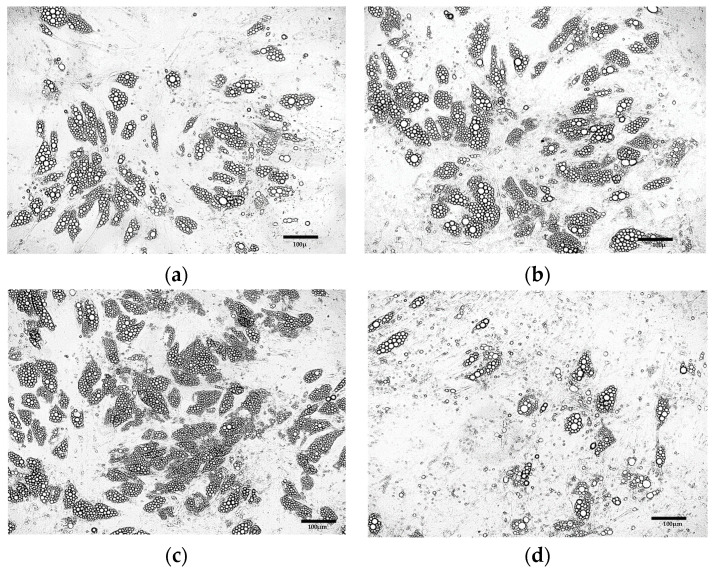
ADSCs cultured for 8 days in differentiation induction medium followed by 13 days in maintenance medium supplemented with 10 µg/mL insulin, 1 μM dexamethasone, and rosiglitazone at the following concentrations: (**a**) 0.2 μM; (**b**) 1 μM; (**c**) 5 μM; (**d**) 10 μM.

Morphological analysis of the cells obtained after ADSC differentiation in media containing various concentrations of rosiglitazone (Figure 6a–d) demonstrated that the optimal rosiglitazone concentration was 5 μM (Figure 6c).

##### Further Optimization of the Differentiation Protocol Using 5 µM Rosiglitazone

**Figure 7:**
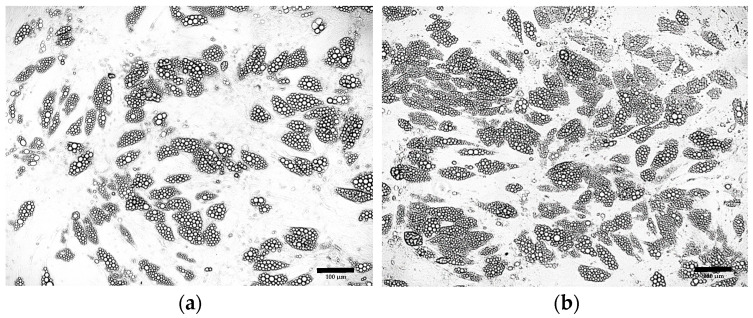
ADSCs cultured according to the optimized protocol supplemented with 5μM rosiglitazone, 1 µM dexamethasone and insulin at concentration: (**a**) 10 μg/mL; (**b**) 20 μg/mL.

Further optimization of the differentiation protocol using 5 µM rosiglitazone confirmed previous findings, indicating that an 8-day induction period and the presence of dexamethasone in the maintenance medium increase the proportion of differentiated cells (Figure 7a). Additionally, increasing the insulin concentration to 20 µg/mL was found to enhance the efficiency of ADSC differentiation and promote lipid droplet formation (Figure 7b).

In contrast, a higher FBS concentration (10%) appeared to inhibit differentiation, likely due to the stimulation of cell proliferation. Moreover, a comparative analysis revealed that both standard DMEM and DMEM/F-12 media support ADSC differentiation into beige adipocytes with comparable efficiency (Supplementary Figure S4).

#### 3.2.2. Optimization of the Differentiation Culture Protocol of ADSCs Towards Beige Adipocytes with Indomethacin

The indomethacin-based protocol led to limited differentiation of ADSCs into beige adipocytes at concentrations ranging from 50 µM to 200 µM. Co-treatment with 1 μM dexamethasone failed to improve differentiation efficiency (Figure S5 Supplement). Morphological analysis revealed a greater number of differentiated cells following the use of adipogenic medium containing rosiglitazone compared to medium supplemented with indomethacin. Furthermore, cells differentiated from ADSCs in the presence of rosiglitazone exhibited a phenotype characteristic of beige adipocytes, as evidenced by the presence of numerous small lipid droplets. Consequently, no further optimization of the differentiation protocol using indomethacin was pursued, and only selected rosiglitazone-containing media variants were used in subsequent experiments.

### 3.3. Oil Red O Staining of Cells Obtained from Differentiation of ADSCs

**Figure 8:**
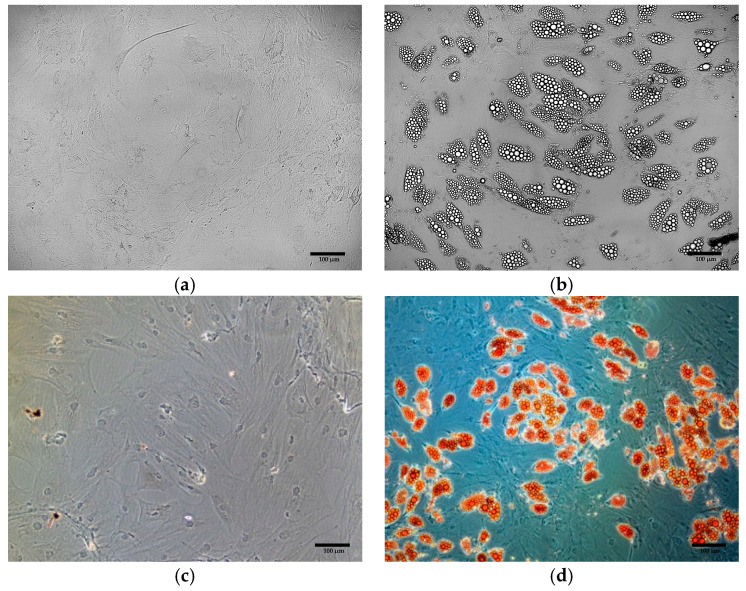
Oil Red O staining of ADSCs: (**a**) ADSCs before differentiation, unstained (**b**) ADSCs after differentiation, unstained (**c**) ADSCs before differentiation, after Oil Red O staining; (**d**) ADSCs after differentiation, after Oil Red O staining.

In differentiated cells, small and abundant lipid droplets, characteristic of beige adipocytes, were observed (Figure 8b). Their presence was confirmed by Oil Red O staining (Figure 8d), whereas no lipid droplets were detected in control cells, either before (Figure 8a), or after staining (Figure 8c).

### 3.4. Quantitative Verification of Differentiation Efficiency

**Figure 9:**
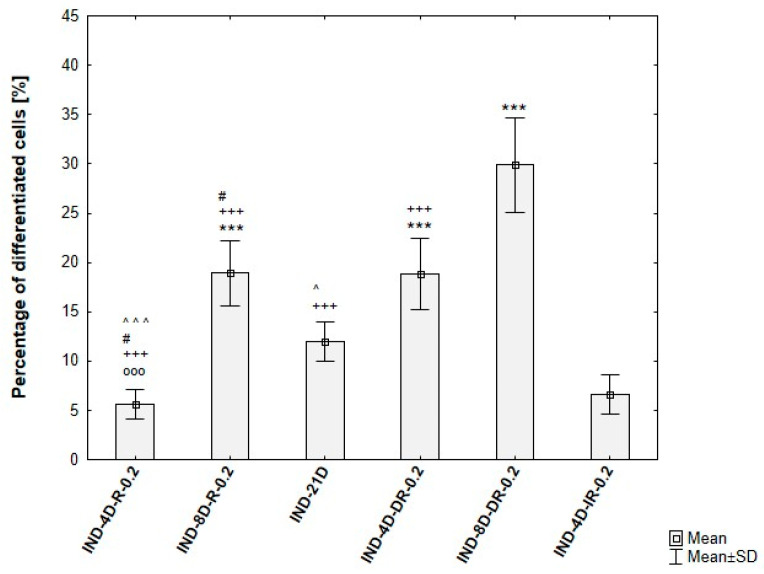
Differentiation efficiency of ADSCs under varying induction durations and maintenance media, based on analysis of microscopic images. IND-4D-R-0.2/IND-8D-R-0.2—ADSCs induced for 4 or 8 days, then maintained with 0.2 µM rosiglitazone + 10 µg/mL insulin, IND-21D—ADSCs induced for 21 days, IND-4D-DR-0.2/IND-8D-DR-0.2—ADSCs induced for 4 or 8 days, then maintained with 0.2 µM rosiglitazone + 10 µg/mL insulin + 1 µM dexamethasone. Statistical analysis was performed using ANOVA followed by Tukey’s post hoc test; ^OOO^ *p* < 0.001 compared to IND-8D-R-0.2, # *p* < 0.05 compared to IND-21D, ^ *p* < 0.05, ^^^ *p* < 0.001 compared to IND-4D-DR-0.2, +++ *p* < 0.001 compared to IND-8D-DR-0.2, *** *p* < 0.001 compared to IND-4D-IR-0.2.

Quantitative analysis of ADSC differentiation confirmed the trends observed during protocol optimization. An induction phase of 4 days resulted in approximately 5% of differentiated cells, while extending the induction to 8 days and including dexamethasone in the maintenance medium increased differentiation efficiency to around 30%. In contrast, prolonged induction (21 days) and IBMX supplementation were associated with pronounced cytotoxic effects, reflected by a high proportion of non-viable cells. These findings are summarized in Figure 9.

**Figure 10:**
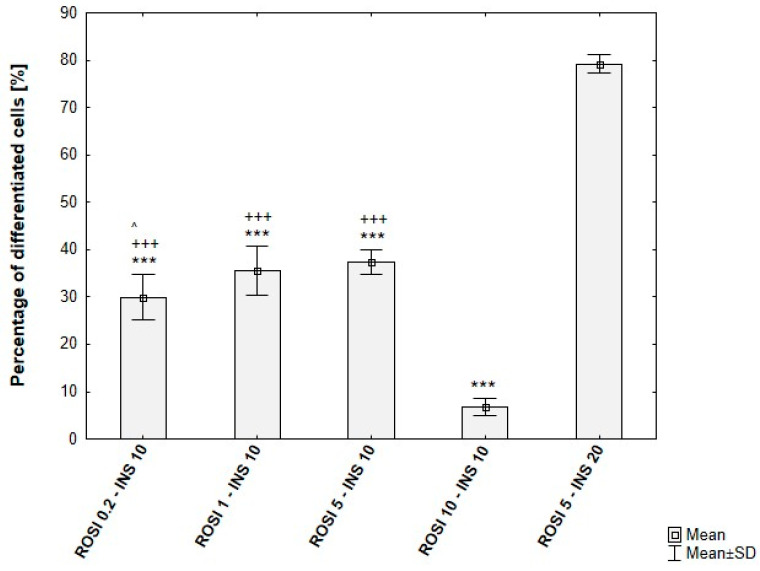
Differentiation efficiency of ADSCs under varying rosiglitazone and insulin concentrations, based on analysis of microscopic images. ROSI 0.2-INS10/ROSI 1-INS10/ROSI 5-INS10/ROSI 10-INS10—ADSCs induced for 8 days, then maintained with rosiglitazone at 0.2, 1, 5, or 10 µM, 10 µg/mL insulin, and 1 µM dexamethasone. ROSI 5-INS20—ADSCs induced for 8 days, then maintained with 5 µM rosiglitazone, 20 µg/mL insulin, and 1 µM dexamethasone. Statistical analysis was performed using ANOVA followed by Tukey’s post hoc test; ^ *p* < 0.05 compared to ROSI 5-INS10, +++ *p* < 0.001 compared to ROSI 10-INS10, *** *p* < 0.001 compared to ROSI 5-INS20.

Among the tested rosiglitazone levels (0.2–10 µM), the 5 µM condition consistently yielded the highest proportion of differentiated cells. Moreover, cultures supplemented with 20 µg/mL insulin exhibited an additional increase in differentiation efficiency (~80%), in agreement with the enhanced lipid droplet formation observed microscopically. The quantitative outcomes of these conditions are presented in Figure 10.

### 3.5. Analysis of UCP1 Gene Expression in ADSCs Undergoing Adipogenic Differentiation

**Figure 11:**
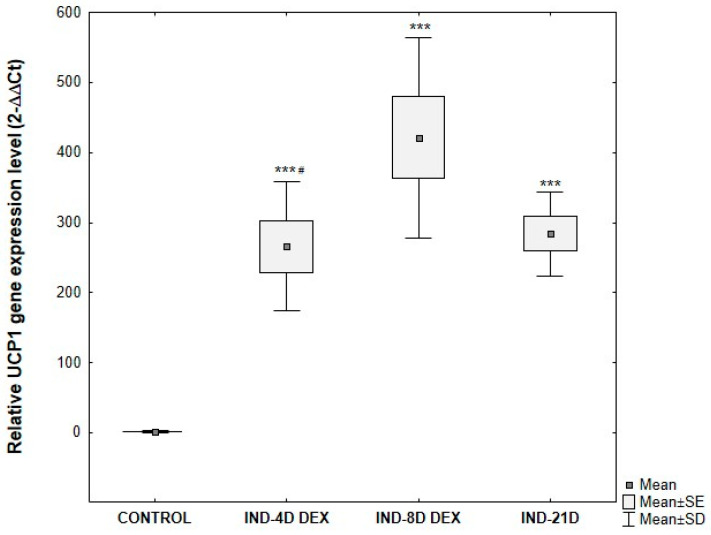
Comparative analysis of changes in relative *UCP1* gene expression at the mRNA level, normalized to the average *GAPDH* gene expression, depending on the duration of the differentiation-inducing culture of ADSCs. ADSCs were cultured for 4 days (IND-4D DEX), 8 days (IND-8D DEX), or 21 days (IND-21D) in induction medium supplemented with 10 μg/mL insulin, 0.2 μM rosiglitazone, 1 μM dexamethasone, and 500 μM IBMX followed by culture without IBMX until day 21 post-T0. Fold change was calculated relative to control using the 2^−ΔΔCT method. Statistical analysis was performed using ANOVA followed by Tukey’s post hoc test, *** *p* < 0.001 compared to the control, # *p* < 0.05 compared to IND-8D DEX.

UCP1 gene expression in ADSCs subjected to differentiating culture conditions showed statistically significant differences compared to control ADSCs cultured under standard conditions. As expected based on morphological analysis, significant differences in UCP1 gene expression were observed depending on the duration of the differentiation-inducing culture. The greatest relative increase in expression compared to control was observed in cells cultured for 8 days in the inducing medium. UCP1 gene expression was significantly higher in these cells compared to those induced for 4 days. There were no statistically significant differences in UCP1 gene expression between ADSCs cultured for 21 days in induction medium and those cultured for 4 days (Figure 11).

**Figure 12:**
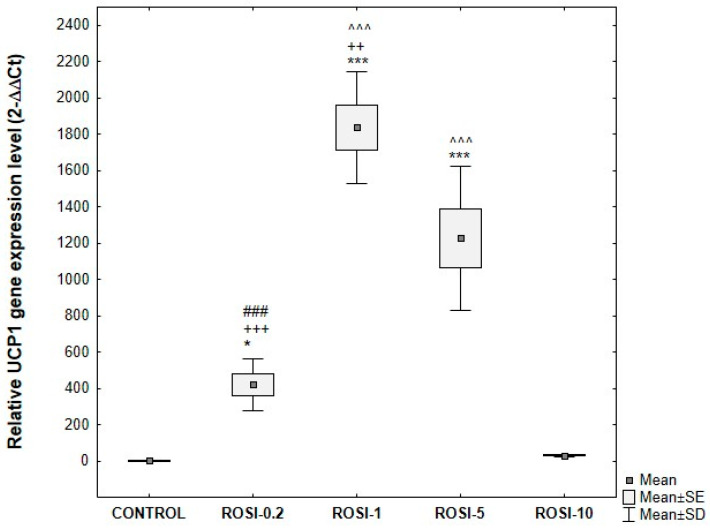
*UCP1* gene expression at the mRNA level, normalized to the average *GAPDH* gene expression in ADSCs exposed to varying concentrations of rosiglitazone, compared with control cells. ADSCs were differentiated for 8 days in induction medium supplemented with 10 μg/mL insulin, 1 μM dexamethasone, 500 μM IBMX, and rosiglitazone at the following concentrations: 0.2 μM (ROSI-0.2), 1 μM (ROSI-1), 5 μM (ROSI-5), and 10 μM (ROSI-10) followed by culture without IBMX until day 21 post-T0. Fold change was calculated relative to control using the 2^−ΔΔCT method. Statistical analysis was performed using ANOVA followed by Tukey’s post hoc test; * *p* < 0.05, *** *p* < 0.001 compared to control, ### *p* < 0.001 compared to ROSI-1, ++ *p* < 0.005, +++ *p* < 0.001 compared to ROSI-5, ^^^ *p* < 0.001 compared to ROSI-10.

The greatest relative increase in UCP1 gene expression compared to control was observed after using differentiation media supplemented with 1 μM and 5 μM rosiglitazone. The mRNA level of UCP1 in cells differentiated with 1 μM and 5 μM rosiglitazone was significantly higher than in cells differentiated with 0.2 μM or 10 μM rosiglitazone (Figure 12).

### 3.6. Semi-Quantitative Analysis of UCP1 Protein by Western Blot in the ADSCs Undergoing Adipogenic Differentiation

**Figure 13:**
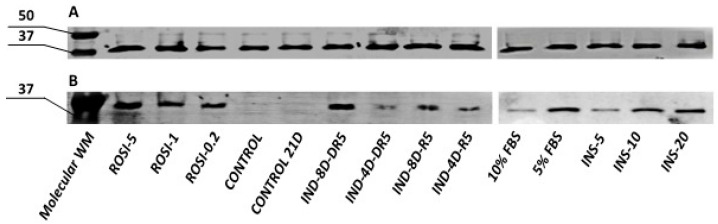
Semi-quantitative analysis of (**A**) GAPDH and (**B**) UCP1 by Western blot in the ADSCs after differentiation towards brown adipose tissue using various protocols. WM- weight marker; ROSI-5, ROSI-1, ROSI-0.2—different concentrations of rosiglitazone (µM); IND-4D/IND-8D—4 or 8 days of induction culture; DR5 –maintenance medium with rosiglitazone and dexamethasone; R5—maintenance medium with rosiglitazone, INS-5, INS-10, INS-20—different concentrations of insulin (μg/mL); 10% FBS, 5% FBS—different concentration of fetal bovine serum in adipogenic medium; CONTROL/CONTROL 21D—ADSCs not subjected to the differentiation process collected immediately after reaching confluency/cultured for three weeks post-confluency.

Semi-quantitative analysis using Western blotting revealed the presence of UCP1 (approximately 33 kDa) in commercial adipose-derived stem cells subjected to differentiation, regardless of the protocol used (Figure 13B). No UCP1 was detected in control cells-ADSCs that were not subjected to differentiation, cultured until confluence (control), or cultured throughout the differentiation period (control 21D). GAPDH protein (approximately 36 kDa) was used as the endogenous control (Figure 13A).

It was estimated that the highest levels of UCP1 protein were found in cells differentiated with medium supplemented with 5 μM rosiglitazone and 20 μg/mL insulin. Furthermore, an 8-day initiating culture and the addition of 1 μM dexamethasone to the differentiation medium resulted in increased UCP1 protein levels compared to cells cultured according to the standard differentiation protocol.

### 3.7. Validation of the Optimized Protocol’s Differentiation Efficiency in Primary ADSCs

#### 3.7.1. Morphological Assessment of Primary ADSCs Differentiated into Beige Adipocytes

**Figure 14:**
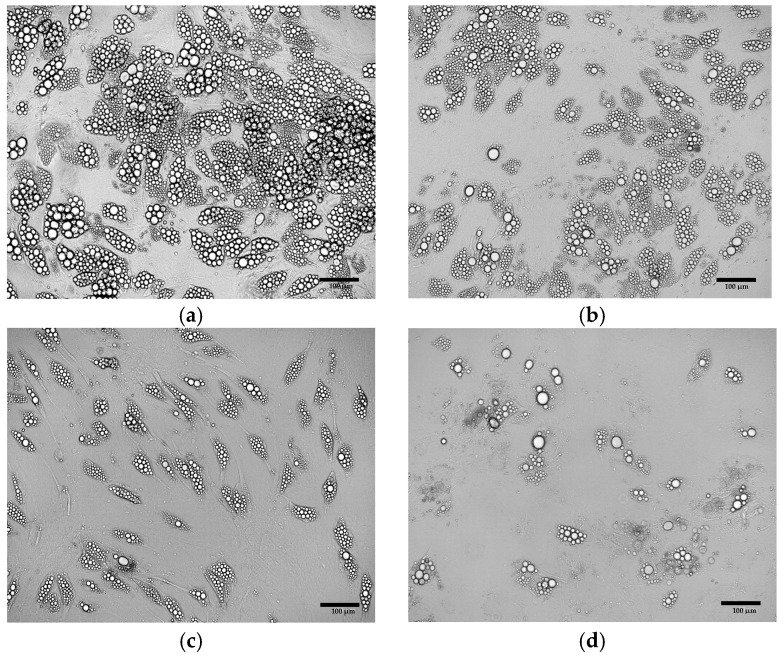
Primary ADSCs after differentiation using the optimized adipogenic protocol. The cells were derived from (**a**) patient no.12; (**b**) patient no.7; (**c**) patient no.15; (**d**) patient no.8.

The culture of primary ADSCs obtained from eight donors, conducted according to the developed protocol, resulted in the formation of lipid droplets, indicating the generation of beige adipocytes (Figure 14a–d). However, the efficiency of the adipogenesis process, estimated from the number of lipid droplets, showed considerable variability between individual donors.

#### 3.7.2. Quantitative Verification of Differentiation Efficiency of Primary ADSCs

**Figure 15:**
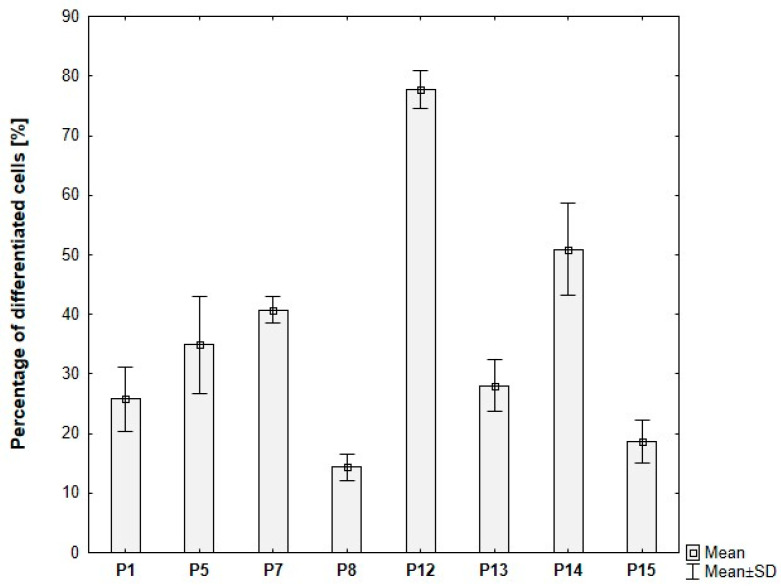
Percentage of differentiated ADSCs for individual donors, based on analysis of microscopic images. ADSCs were differentiated for 8 days in induction medium supplemented with 20 μg/mL insulin, 1 μM dexamethasone, 500 μM IBMX, and 5 μM rosiglitazone, followed by culture without IBMX until day 21 post-T0.

Donor-dependent variability in ADSC differentiation was observed. The proportion of differentiated cells ranging from 15% to approximately 80% across different donors, despite identical induction protocols and culture conditions (Figure 15).

#### 3.7.3. Analysis of UCP1 Gene Expression in Primary ADSCs Undergoing Adipogenic Differentiation

**Figure 16:**
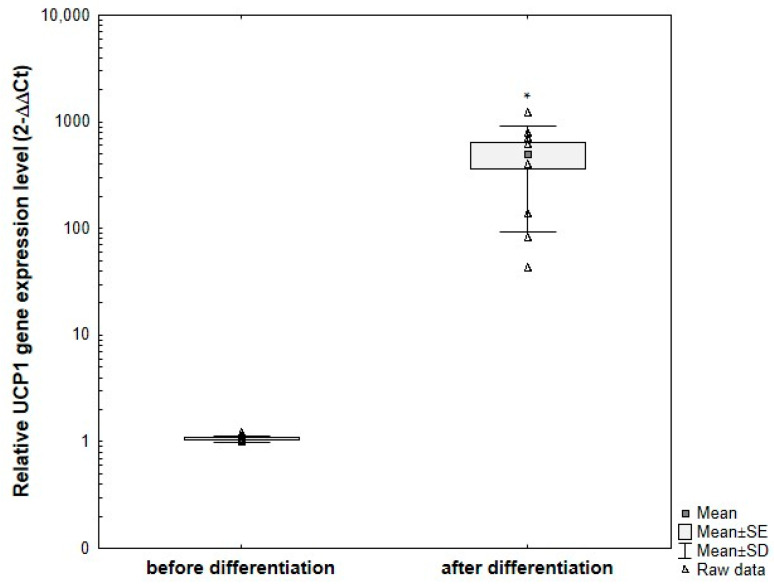
Comparative analysis of changes in relative *UCP1* gene expression at the mRNA level, normalized to the average *GAPDH* gene expression, in primary ADSCs obtained from different adults and subjected to differentiation using the optimized protocol, compared to undifferentiated control cells. ADSCs were differentiated for 8 days in induction medium supplemented with 20 μg/mL insulin, 1 μM dexamethasone, 500 μM IBMX, and 5 μM rosiglitazone, followed by culture without IBMX until day 21 post-T0. Fold change was calculated relative to control using the 2^−ΔΔCT method. Statistical analysis was performed using ANOVA followed by Tukey’s post hoc test, * *p* < 0.05.

*UCP1* gene expression in primary ADSCs obtained from donors and subjected to the optimized differentiation protocol showed statistically significant differences compared to undifferentiated ADSCs cultured under standard conditions (Figure 16). As expected based on protein expression analysis, the level of *UCP1* gene expression varied between subjects. In all analyzed samples, a relative increase in UCP1 expression was observed after differentiation compared to the corresponding undifferentiated controls.

#### 3.7.4. Semi-Quantitative Analysis of UCP1 Protein by Western Blot in the Primary ADSCs Undergoing Adipogenic Differentiation

**Figure 17:**
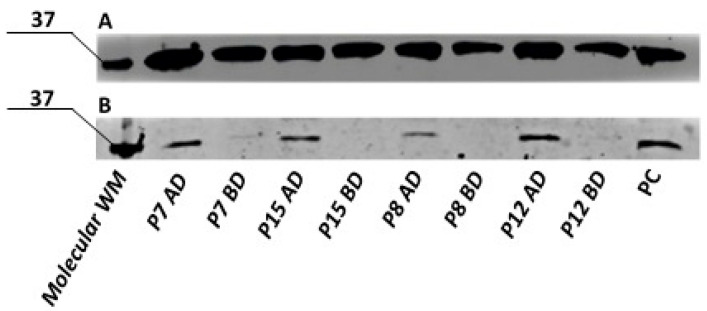
Semi-quantitative analysis of GAPDH (**A**) and UCP1 (**B**) by Western blot in the primary ADSCs collected from different donors, after differentiation towards brown adipose tissue using optimized protocol. WM- molecular weight marker; P7,P15,P58,P12—primary human ADSCs from different donors; AD—after differentiation, BD—before differentiation; PC—positive control.

In primary human ADSCs, no UCP1 protein was detected in undifferentiated cells. However, cells subjected to the optimized differentiation protocol exhibited UCP1 protein expression, with expression levels varying between individuals. Representative samples from four subjects are shown in Figure 17.

### 3.8. Summary

Based on the morphological assessment of ADSCs undergoing adipogenic differentiation, along with Oil Red O staining, analysis of *UCP1* gene expression at the mRNA level via RT-qPCR, and semi-quantitative evaluation of UCP1 protein levels using Western blot, the following conclusions were drawn:

- Rosiglitazone is a more effective inducer of beige adipocyte differentiation from ADSCs compared to indomethacin;
- The optimal differentiation conditions include 5 μM rosiglitazone and 20 μg/mL insulin in the culture medium;
- Extending the induction phase in induction medium to 8 days enhances the efficiency of ADSC differentiation into adipocytes; however, further extension of this period may lead to cytotoxic effects;
- Enrichment of the adipogenic medium with 1 μM dexamethasone throughout the entire differentiation period enhances adipogenic differentiation;
- ADSCs do not undergo spontaneous differentiation into beige adipocytes during long-term (three-week) post-confluent culture.

According to these observations, a new differentiation protocol was developed for inducing beige adipocyte formation from ADSCs. A comparison between the newly established protocol and the original base protocol selected at the beginning of the experiment (based on literature data) is presented in Table 2. The optimized protocol is effective in inducing differentiation of primary human ADSCs obtained from different adult donors, although its efficiency varies between individuals.

## 4. Discussion

In recent years, brown adipose tissue has gained significant attention as a subject of intensive research, particularly in the context of therapeutic strategies for metabolic diseases such as type 2 diabetes and obesity, as well as in the prevention of cardiometabolic complications. Research is focused both on the activation of endogenous BAT and on the browning process, which involves the formation of beige adipocytes. These beige adipocytes can arise in vivo from white adipose tissue (WAT) and can also be generated through the differentiation of adipose-derived stem cells.

Standard protocols for inducing beige adipocytes from ADSCs typically follow a two-step procedure: an initial 4-day culture in an induction medium containing rosiglitazone (or indomethacin), dexamethasone, IBMX, and insulin, followed by a maintenance phase in a medium without dexamethasone and IBMX continued for up to 16–21 days. ADSCs initially differentiate into white adipocytes; however, prolonged exposure to differentiating factors promotes their conversion into beige adipocytes, characterized by numerous small intracellular lipid droplets [17,18].

Although widely used, the above protocol varies significantly across studies in terms of culture duration, medium composition, and factor concentrations, due to the lack of a standardized method, which can lead to substantial variability in outcomes across laboratories. Most research relies on commercial or animal-derived MSCs, with few studies evaluating human donor-derived MSCs, which further hampers the applicability of the findings to clinical medicine.

In the present study, an optimization of the differentiation culture protocol for adipose-derived stem cells (ADSCs) toward beige adipocytes was conducted. Over 30 protocol variants were evaluated, varying in the duration of the induction phase, media composition, and concentrations of differentiation factors. The efficiency of cellular differentiation was assessed based on morphological analysis (image-based quantification of differentiated cells), intracellular lipid droplet staining using Oil Red O, and the evaluation of *UCP1* gene expression—a key marker of brown adipocyte—at the transcript level by RT-qPCR and at the protein level using Western blot. The final stage of the study involved evaluating the effectiveness of the optimized protocol in primary human ADSCs from eight independent donors.

Due to reports in the literature regarding spontaneous differentiation of ADSCs, this study compared ADSCs cultured for three weeks post-confluency with cells collected immediately after reaching confluency. Under the conditions tested, no spontaneous adipogenic differentiation was observed—there were no characteristic morphological changes nor expression of the *UCP1* gene at the mRNA or protein levels. The literature presents conflicting data: Yang et al. [30], and Dudakovic et al. [32] reported the possibility of spontaneous differentiation of ADSCs into different cell types, although Yang et al. [30] noted that this phenomenon disappears after the first passage. Conversely, Roxburgh et al. [33] did not confirm spontaneous adipogenesis in ADSCs. This work examines also the effects of selected factors on adipogenesis. It was demonstrated that none of the individual factors—indomethacin, rosiglitazone, insulin, IBMX, or dexamethasone—when applied alone, initiated differentiation of ADSCs into beige adipocytes. Similar results were reported by Contador et al. [34] for BM-MSCs.

Many protocols for MSC differentiation into adipocytes rely on the use of indomethacin at concentrations ranging from 50 μM to 200 μM as the primary differentiation agent. Alternatively, other protocols employ rosiglitazone, with its optimal concentration in the medium varying between 0.1 μM and 10 μM according to different adipogenic schemes (Supplementary Table S1). In this study, the effectiveness of ADSC differentiation protocols using various concentrations of indomethacin and rosiglitazone was compared. Based on microscopic observations and Oil Red O staining, it was demonstrated that media containing rosiglitazone resulted in a higher number of adipocytes compared to media with indomethacin, regardless of the concentration applied. Therefore, the rosiglitazone-based protocol was selected for further optimization.

In the current research, it was demonstrated that differentiation of ADSCs into adipocytes requires the presence of rosiglitazone in the culture medium. The combination of dexamethasone, IBMX, and insulin—without rosiglitazone—did not initiate the differentiation process. Similar findings were reported by Kim et al. [35], who showed that in the absence of rosiglitazone, only a small fraction of BM-MSCs underwent differentiation, whereas its presence significantly increased differentiation efficiency in a concentration-dependent manner (within the range of 0.1–10 μM). However, other studies have demonstrated that while rosiglitazone may enhance adipogenic differentiation, it is not strictly required for the differentiation of certain types of MSCs [36,37], suggesting that the regulatory mechanisms of adipogenesis may differ between MSC populations.

In the next phase of the study, it was confirmed that effective induction of adipogenesis requires the simultaneous presence of dexamethasone, IBMX, and insulin along with rosiglitazone in the culture medium. Omission of any of these components resulted in a marked reduction in the number of differentiated cells. These findings are consistent with the results reported by Contador et al. [34], who demonstrated that rosiglitazone treatment alone was insufficient to induce the formation of functional adipocytes from human MSCs. According to Nuttall et al. [36], BM-MSCs are insulin-independent, whereas insulin serves as a key differentiating factor for non-marrow-derived cells. Our study further confirmed the crucial role of insulin in ADSC differentiation—the increase in insulin concentration correlated with a higher number of adipocytes formed, with the most optimal effects observed at 20 μg/mL. The agreement between our findings and the literature suggests a synergistic interaction among rosiglitazone, insulin, IBMX, and dexamethasone, which collectively create optimal conditions for an efficient differentiation of ADSCs into adipocytes.

This work also aimed to assess the impact of extending the induction phase, which normally lasts approximately 4 days, on the efficiency of ADSC differentiation. Based on cell morphology, Oil Red O staining, as well as RT-qPCR and Western blot analyses, we demonstrated that prolonging the induction culture from 4 to 8 days resulted in an increased number of differentiated cells, a higher accumulation of intracellular lipid droplets, and elevated expression of the *UCP1* gene at both mRNA and protein levels. However, maintaining the induction medium throughout the entire 21-day differentiation period led to progressive cell death, likely due to prolonged exposure to IBMX. Similar findings were reported by Lee et al. [38], who showed that extending the induction culture time from 3 to 7 days or longer increased the differentiation efficiency of preadipocytes from approximately 30–70% to over 80%. ADSCs represent a heterogeneous cell population. It is possible that within this group there are subpopulations with varying differentiation capacities; some of these may, in turn, require a longer induction period to initiate differentiation, which could explain the observed results.

A novel approach applied in this study was the supplementation of the culture medium with dexamethasone throughout the entire differentiation period. The results demonstrated that this strategy significantly enhanced the efficiency of ADSC differentiation into beige adipocytes. The consistency of these observations with reports from other authors suggests that prolonged exposure to dexamethasone in the culture environment may represent a universal mechanism supporting MSC differentiation. For instance, Oshina et al. [39] showed that treating human BM-MSCs with dexamethasone during the entire culture period prior to differentiation increased their multilineage differentiation potential, including osteogenic, chondrogenic, and adipogenic pathways. This effect may be related to dexamethasone’s ability to induce apoptosis, which is one mechanism leading to the redistribution and alteration of cell subpopulations. Dexamethasone may promote the proliferation of cells with high differentiation potential while selectively eliminating cells with lower differentiation capacity [39]. Similarly, Pantoja et al. [37] observed that the efficiency of differentiation of 3T3-L1 preadipocytes into beige adipocytes was higher when cells were first treated with dexamethasone followed by IBMX, compared to the reverse sequence of treatment.

The investigation of various rosiglitazone concentrations in the culture medium revealed that its effect on the efficiency of ADSC differentiation may be dose-dependent, both at the protein level and in terms of cellular phenotype. The most effective concentration was 5 μM, as confirmed by semi-quantitative Western blot analysis, microscopic observations, and Oil Red O staining of lipid droplets. Although the mRNA expression level of the *UCP1* gene was higher in cells differentiated with 1 μM rosiglitazone compared to those treated with 5 μM, it should be noted that mRNA levels do not always correlate with protein levels or cellular phenotype. According to the literature, the correlation between mRNA expression levels and corresponding protein amounts is generally moderate. These discrepancies may result from both biological and technical factors, which should be taken into account when interpreting the results [40,41].

In our study, no significant differences were observed in the efficiency of adipogenic differentiation of ADSCs cultured in either DMEM or DMEM/F-12, suggesting that both media are equally effective in inducing adipogenesis in these cells. Conversely, increasing the FBS concentration from 5% to 10% led to a decrease in the proportion of differentiated cells. This may indicate that higher FBS concentrations influence other cellular mechanisms, such as enhanced proliferation, which in turn inhibits adipogenic differentiation. Our observations are consistent with findings by Lee et al. [38], who also reported that increasing FBS concentration in the medium reduces the efficiency of preadipocytes differentiation into adipocytes.

In contrast to more complex differentiation protocols, such as the B-8 medium protocol [12], which relies on multiple recombinant growth factors, inhibitors, and trace element mixtures, our study aimed to develop a simplified and cost-effective approach based on the systematic optimization of classical adipogenic inducers (rosiglitazone, indomethacin, dexamethasone, insulin, and IBMX). This strategy allowed for reproducible differentiation across multiple donor-derived ADSC lines while maintaining experimental accessibility and scalability for future mechanistic studies. Importantly, by omitting exogenous cytokines and growth factors such as BMP7 or FGF2, our protocol preserves the endogenous secretion profile of ADSCs and beige adipocytes, making it particularly suitable for studies of autocrine and paracrine signaling. Such simplification facilitates the use of this model in a wider range of laboratories and provides a standardized baseline for future research on beige adipogenesis and its regulation.

During differentiation culture, only a subset of cells undergo differentiation, while a portion remains undifferentiated. A possible reason for this is the previously described heterogeneity of ADSCs, characterized by the presence of multiple subpopulations differing in marker expression, such as CD105 or CD146, which may exhibit varying susceptibility to the applied differentiation factors. Therefore, it should be noted that the measured gene expression at the mRNA and protein levels represents an average, reflecting the expression in both differentiated cells and undifferentiated stem cells. As a result, the overall gene expression is reduced compared to the actual expression level in the differentiated cell population.

Throughout the differentiation culture, cells were found to undergo adipogenic differentiation, forming characteristic clusters. In the vicinity of differentiated cells, new cells continuously appeared and gradually underwent differentiation, while large areas of other cells showed no signs of differentiation. This phenomenon may indicate paracrine properties of ADSCs. It is likely that differentiated cells secrete factors that influence the induction of differentiation in neighboring cells. Li et al. [42] demonstrated that donor MSCs differentiating towards the osteogenic lineage secrete factors that subsequently induce differentiation and migration of endogenous MSCs. Recently, numerous studies have focused on the paracrine activity of MSCs, which involves secretion of various factors such as growth factors, cytokines, and chemokines [31,43], as well as the release of exosomes containing proteins, lipids, and RNA [44]. This aspect may serve as a starting point for further analyses.

Another innovative aspect of this study was the use of primary human ADSCs directly isolated from adipose tissue of human donors. To date, only a limited number of studies have utilized such cells, resulting in a scarcity of reliable data regarding the efficacy of established adipogenic protocols in these cells. Furthermore, there is a lack of detailed analyses that consider inter-individual variability, which may significantly affect the efficiency of the differentiation process. Understanding the factors influencing adipogenesis is crucial for developing optimized ADSC differentiation methods tailored to individual patient characteristics. This knowledge could pave the way for future applications of ADSCs in both autologous therapies and treatments involving agents that induce ADSC differentiation or WAT conversion toward brown adipose tissue in vivo. Therefore, the final stage of the study involved evaluating the efficiency of beige adipocytes tissue differentiation using primary ADSCs obtained from eight donors, based on the protocol optimized in the initial phase of the experiment. The primary ADSCs exhibited minimal expression of the CD45 antigen, while showing high expression of surface markers CD90 and CD105. In contrast, expression of the CD146 surface marker was detected in less than 20% of the cells (results presented in Wystrychowski et al., 2024) [31].

The protocol developed in this study proved to be effective—ADSCs derived from all patients demonstrated the ability to differentiate into beige adipocytes. However, significant differences were observed in the percentage of differentiated cells across individual samples, ranging from approximately 15% to 80% of the total cell population. These differences may stem from individual biological characteristics of the donors and will be the subject of further research.

## 5. Conclusions

In the present study, an optimized protocol for the differentiation of adipose-derived stem cells (ADSCs) into beige adipocytes was established and demonstrated to be effective for ADSCs from eight human donors. Notably, the efficiency of differentiation varied among individual patient samples, likely due to inter-individual variability or other biological factors. This protocol provides a simple, reproducible, and biologically driven framework that preserves endogenous cytokine secretion, enabling studies of autocrine and paracrine signaling. Its flexibility and scalability make it suitable for metabolic assays, gene expression analyses, and evaluation of donor variability, offering a robust and accessible in vitro model for both basic and translational research in beige adipose tissue biology. Our findings also highlight the relevance of personalized approaches in metabolic research.

Beige adipocytes, similar to classical brown adipose tissue (BAT), possess thermogenic capacity driven by the expression of uncoupling protein 1 (UCP1), which uncouples oxidative phosphorylation in mitochondria. This process leads to increased utilization of energy substrates—such as glucose and fatty acids—resulting in improved glucose homeostasis and enhanced insulin sensitivity [3,4,5,6,7,8]. Importantly, the presence of active beige adipocytes may act synergistically with BAT, not only through local paracrine effects but also via systemic endocrine signaling. BAT-derived cytokines, often referred to as “batokines” can stimulate the recruitment and activation of BAT as well as promote further “browning” of white adipose tissue [45]. As a result, the overall energy expenditure of the organism is increased, which in patients with diabetes may contribute to improved glycemic control, weight reduction, and enhanced lipid metabolism.

A better understanding of the regulatory mechanisms underlying beige adipocyte formation is crucial. The development of a unified differentiation protocol and standardized in vitro models for studying adipogenesis and beige fat metabolism is particularly important. Identifying the key factors that drive ADSCs toward a beige adipocyte phenotype may ultimately facilitate the development of safe and effective therapies aimed at activating BAT in vivo.

Future research should focus on validating the proposed protocol in a larger and more diverse patient cohort, as well as determining whether metabolic diseases such as obesity or diabetes impair the ability of ADSCs to differentiate into beige adipocytes. In addition, the cytokine profile of patient-derived ADSCs will be analyzed before and after differentiation to gain deeper insights into their metabolic activity and intercellular signaling potential.

## Figures and Tables

**Table 1:** Clinical and biochemical characteristics of the study participants.

	Age	BMI	Glu	Ins	HbA1c	HOMA-IR	Cr	TC	HDL	LDL	TG	CRP
Mean	43	27.3	82.7	7.1	5.1	1.4	0.8	201.2	65.7	115.6	98.5	2.34
SD	12	4.4	7.6	4.2	0.2	0.8	0.1	39.6	13.5	29.0	70.0	3.11
Min	28	21.5	65	2.7	4.8	0.5	0.7	166.0	48.0	69.0	49.0	0.02
Max	63	36.0	88.3	15.4	5.4	3.0	1.0	267.7	87.0	150.9	252.5	8.1

Data are presented as mean ± standard deviation (SD) with minimum and maximum values. The study group included 8 donors—5 females and 3 males. Abbreviations: Age—age (years); BMI—body mass index (kg/m^2^); Glu—glucose (mg/dL); Ins—insulin (µIU/mL); HbA1c—glycated hemoglobin (%); HOMA-IR—homeostatic model assessment of insulin resistance; Cr—creatinine (mg/dL); TC—total cholesterol (mg/dL); HDL—high-density lipoprotein cholesterol (mg/dL); LDL—low-density lipoprotein cholesterol (mg/dL); TG—triglyceride (mg/dL); CRP—C-reactive protein (mg/L).

**Table 2:** Comparison of the differentiation protocol selected for optimization based on literature data with the protocol optimized during the course of the experiment.

	Induction Medium		Maintenance Medium	
	Composition	Duration	Composition	Duration
Baseline Protocol	0.2–10 μM rosiglitazone 1 μM dexamethasone 500 μM IBMX 10 μg/mL insulin	4 days	0.2–10 μM rosiglitazone 10 μg/mL insulin	12–17 days
Optimized Protocol	5 μM rosiglitazone 1 μM dexamethasone 500 μM IBMX 20 μg/mL insulin	8 days	5 μ M rosiglitazone 1 μ M dexamethasone 20 μ g/mL insulin	13 days

Modifications introduced during optimization are highlighted in red.

## Data Availability

The original contributions presented in this study are included in the article/Supplementary Materials. Further inquiries can be directed to the corresponding author.

## Supplementary Materials

The following supporting information can be downloaded at: https://www.mdpi.com/article/10.3390/cells15010054/s1, Figure S1. ADSCs cultured for 21 days in DMEM/F12 medium with or without differentiation factors; Figure S2. ADSCs cultured for 21 days in the simultaneous presence of different differentiation factors; Figure S3. ADSCs cultured for 21 days: 4 days in induction medium followed by 17 days in differentiation medium supplemented with insulin at the indicated concentration: (a) 0 μg/mL; (b) 1 μg/mL; (c) 5 μg/mL; (d) 10 μg/mL; Figure S4. ADSCs cultured according to the optimized protocol in: (a) DMEM supplemented with 5% FBS; (b) DMEM/F-12 supplemented with 5% FBS; and (c) DMEM/F-12 supplemented with 10% FBS; Figure S5. ADSCs differentiated for 4 days in induction medium containing 10 µg/mL insulin, 1 µM dexamethasone, 500 µM IBMX, and indomethacin at a concentration of: (a) 0 µM; (b) 50 µM; (c) 100 µM; (d) 200 µM, followed by 17 days in maintenance medium; Table S1. Comparison of differentiation factor concentrations used in selected protocols based on literature data; Table S2. Detailed description of the protocols and media compositions used in the present study.

## Abbreviations

The following abbreviations are used in this manuscript:

ADSCs	Adipose-Derived Stem Cells
BAT	Brown Adipose Tissue
BM-MSCs	Bone Marrow Mesenchymal Stem Cells
DEXA	Dexamethasone
DMEM	Dulbecco’s Modified Eagle Medium
DMSO	Dimethyl Sulfoxide
FBS	Fetal Bovine Serum
GAPDH	Glyceraldehyde-3-Phosphate Dehydrogenase
IBMX	3-Isobutyl-1-Methylxanthine
Il	Interleukin
IND	Induction
INDO	Indomethacin
INS	Insulin
MSCs	Mesenchymal Stem Cells
PCOS	Polycystic Ovary Syndrome
ROSI	Rosiglitazone
RT-qPCR	Reverse Transcription Quantitative Polymerase Chain Reaction
TNF-A	Tumor Necrosis Factor-Alpha
UCP1	Uncoupling Protein 1
WAT	White Adipose Tissue
