# Supplementary material for: Toward Efficient Beige Adipogenesis: Protocol Optimization Using Adipose-Derived Stem Cells
Source: Cells. 2025 Dec 28;15(1):54. doi: 10.3390/cells15010054 (PMC12785445; doi:10.3390/cells15010054)
Supplement: Supplementary file 1 [file cells-15-00054-s001.zip › Supplementary Figures.pdf]

Supplementary Materials: Figures

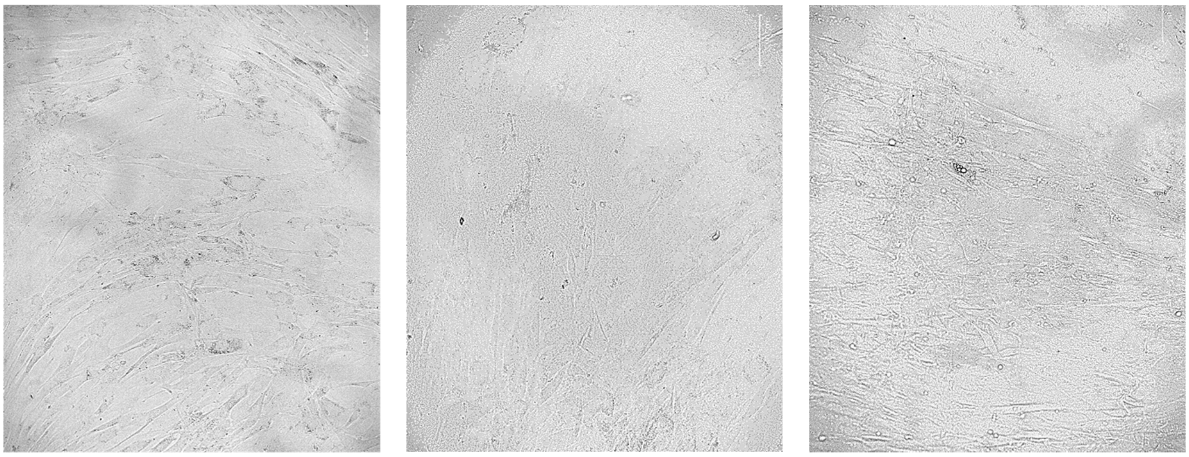

(a) **(b)** **(c)**

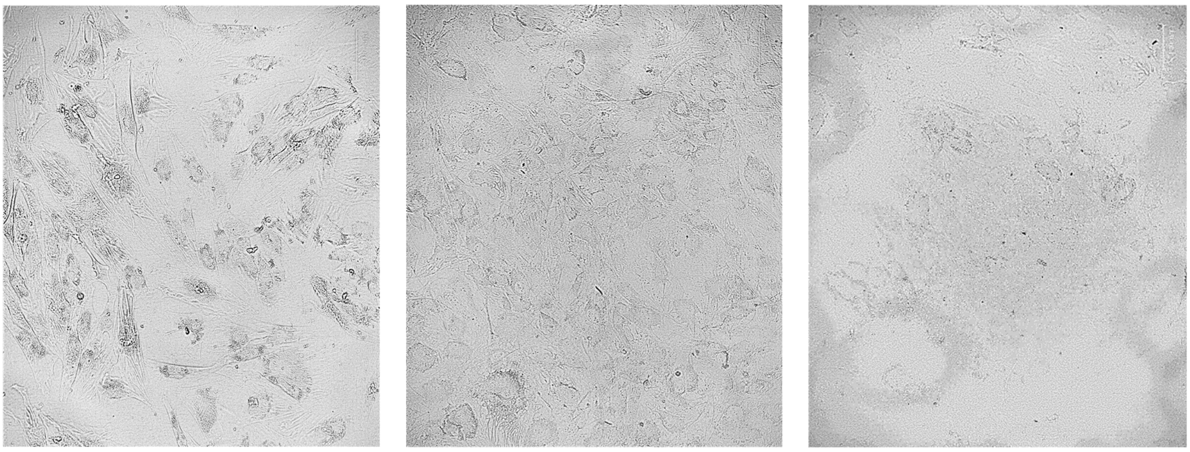

(d) **(e)** **(f)**

Figure S1. Effect of individual differentiation factors on ADSC morphology after 21 days of culture. ADSCs were cultured for 21 days in DMEM/F12 medium under different conditions: (a) no supplements (b) only 10 μg/mL insulin; (c) only 1 μM dexamethasone; (d) only 500 μM 3-isobutyl-1-methylxanthine (IBMX); (e) only 0.2 μM rosiglitazone; (f) only100 μM indomethacin.

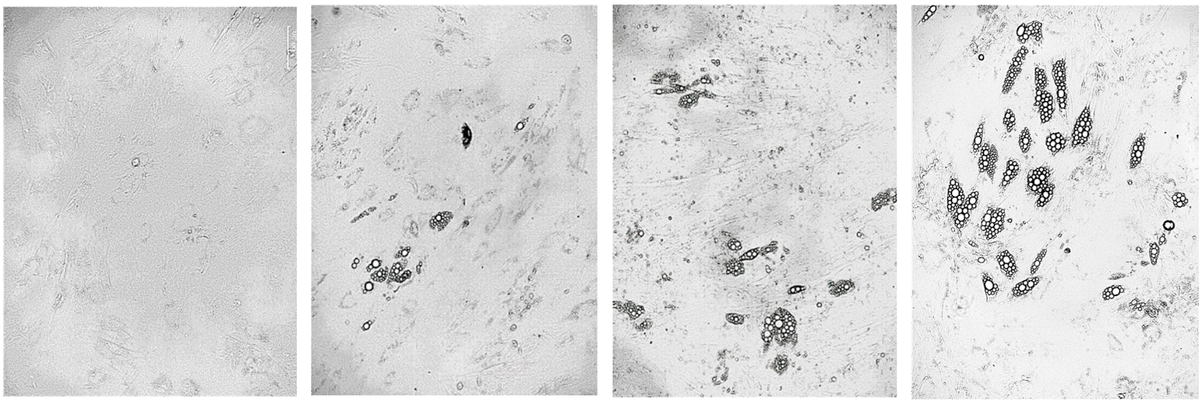

(a) **(b)** **(c)** **(d)**

Figure S2. ADSCs cultured for 21 days in the simultaneous presence of different differentiation factors: (a) dexamethasone and IBMX; (b) rosiglitazone and IBMX; (c) rosiglitazone and dexamethasone; (d) ADSCs cultured for 4 days with rosiglitazone, dexamethasone, and IBMX, followed by 17 days with rosiglitazone alone.

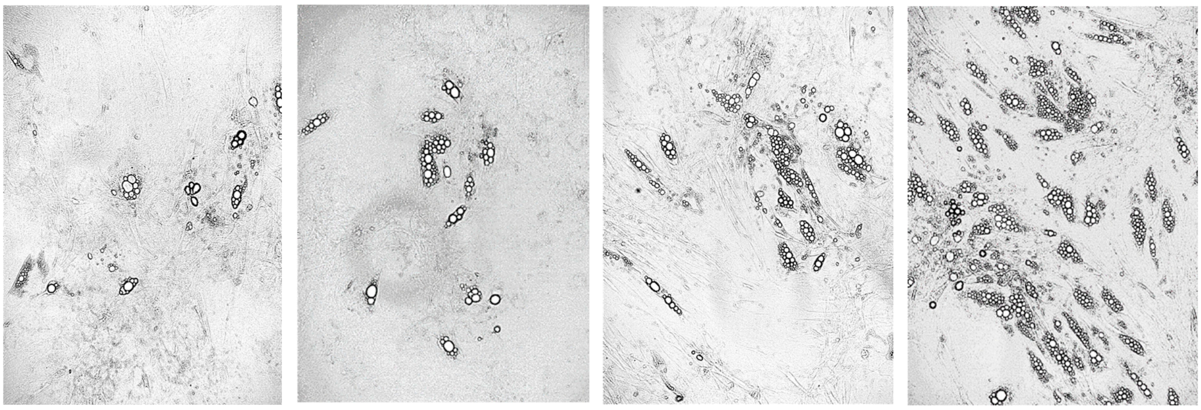

(a) **(b)** **(c)** **(d)**

Figure S3. ADSCs cultured for 21 days: 4 days in induction medium followed by 17 days in differentiation medium supplemented with insulin at the indicated concentration: (a) 0 μg/ml; (b) 1 μg/ml; (c) 5 μg/ml; (d) 10 μg/ml.

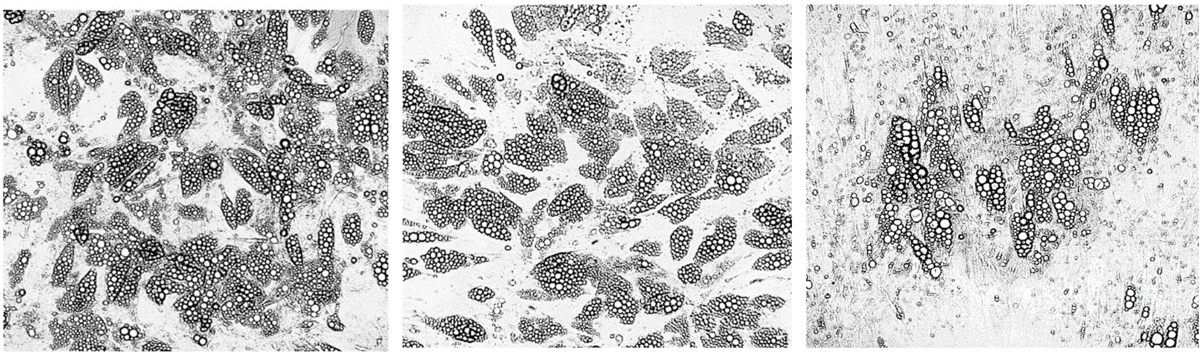

(a) **(b)** **(c)**

Figure S4. ADSCs cultured according to the optimized protocol in: (a) DMEM supplemented with 5% FBS; (b) DMEM/F-12 supplemented with 5% FBS; and (c) DMEM/F-12 supplemented with 10% FBS.

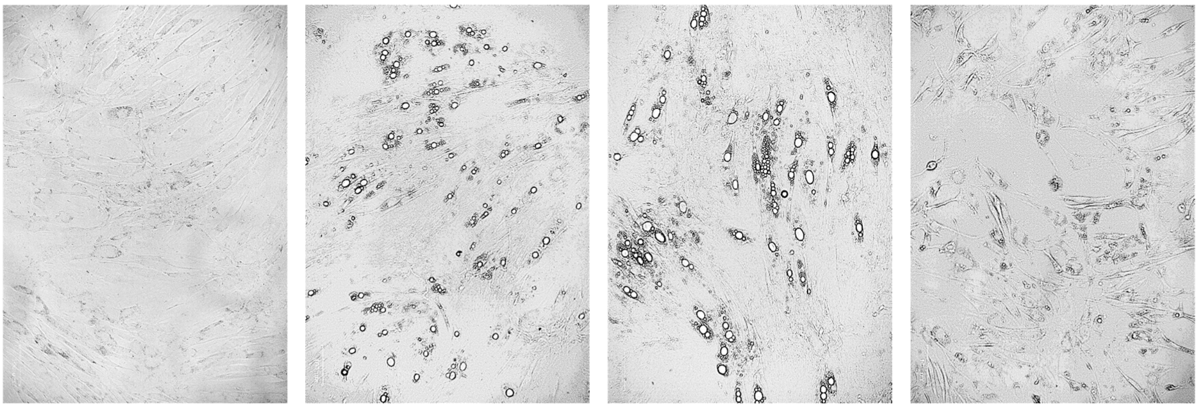

(a) **(b)** **(c)** **(d)**

Figure S5. ADSCs differentiated for 4 days in induction medium containing 10 μg/mL insulin, 1 μM dexamethasone, 500 μM IBMX, and indomethacin at a concentration of: (a) 0 μM; (b) 50 μM; (c) 100 μM; (d) 200 μM, followed by 17 days in maintenance medium.
