# Supplementary material for: Toward Efficient Beige Adipogenesis: Protocol Optimization Using Adipose-Derived Stem Cells
Source: Cells. 2025 Dec 28;15(1):54. doi: 10.3390/cells15010054 (PMC12785445; doi:10.3390/cells15010054)
Supplement: Supplementary file 1 [file cells-15-00054-s001.zip › Supplementary Tables.pdf]

Supplementary Materials: Tables

Table S1. Comparison of differentiation factor concentrations used in selected protocols based on literature data.

Publication		Differentiation factors				
**No.**	**Author**	**IBMX [μM]**	**DEXA [μM]**	**INS**	**INDO [μM]**	**ROSI [μM]**
1.	Lee (15)	187	1	1 μM	200	-
2.	Zimmerlin (16)	0.5	1	10 μg/ml	60	-
3.	Park (17)	500	1	10 μM	200	-
4.	Ghosh (18)	500	1	10 μM	200	-
5.	Kalinina (19)	500	1	10 μM	200	-
6.	Barbagallo (20)	500	0.1	0.1 μM	60	-
7.	Lauvrud (21)	500	1	10 μg/ml	100	-
8.	Yu (22)	500	1	1 μM	-	5
9.	Pisani (23)	500	1	0.85 μM	-	0.1
10.	Elabd (24)	500	1	0.85 μM	-	0.02 – 0.5
11.	Mohsen-Kanson (25)	500	0.25	1 μg/ml	-	1
12.	Kim (26)	500	1	10 μg/ml	-	10
13.	Wu (27)	250	1	1 μM	-	2
14.	Carey (28)	500	25	1 μM	0.250	1
15.	Aune (29)	500	2 ug/ml	5 μg/ml	125	0.5
16.	Yang (30)	500	1	10 μM	200	-

Table S2. Detailed description of the protocols and media compositions used in the present study.

No.	*Name*	INDUCTION CULTURE		MAINTENANCE CULTURE	
		**Time**	**Composition**	**Time**	**Composition**
		**Evaluation of the influence of various factors on ADSC culture**			
**1.**	**CONTROL**	21 days	**-**	-	-
**2.**	**DEXA**		1 μM dexamethasone		
**3.**	**ROSI**		0.2 μM rosiglitazone		
**4.**	**IBMX**		500 μM IBMX		
**5.**	**INSULIN**		10 μg/ml insulin		
**6.**	**INDOMETHACIN**		100 μM indomethacin		
		**Optimization of the induction medium composition**			
**7.**	**DEXA + IBMX**	21 days	1μM dexamethasone, 500 μM IBMX, 10 μg/ml insulin	-	-
**8.**	**ROSI + IBMX**		0.2 μM rosiglitazone, 500 μM IBMX, 10 μg/ml insulin		
**9.**	**ROSI + DEX**		0.2 μM rosiglitazone, 1 μM dexamethasone, 10 μg/ml insulin		
		**Optimization of the induction culture duration**			
**10.**	**IND-0D**	0 days	-	21 days	0.2 μM rosiglitazone, 10 μg/ml insulin
**11.**	**IND-4D**	4 days	0.2 μM rosiglitazone, 500 μM IBMX 1 μM dexamethasone insulin 10 μg/ml	17 days	
**12.**	**IND- 8D**	8 days		13 days	
**13.**	**IND- 21D**	21 days		0 days	-

	**Optimization of insulin concentration**				
**14.**	**0 μg/ml INS**	**4 days**	0.2 μM rosiglitazone, 500 μM IBMX 1 μM dexamethasone,	17 days	0.2 μM rosiglitazone, 0 μg/ml insulin
**15.**	**1 μg/ml INS**		0.2 μM rosiglitazone, 500 μM IBMX 1 μM dexamethasone, 1 μg/ml insulin		0.2 μM rosiglitazone, 1 μg/ml insulin
**16.**	**5 μg/ml INS**		0.2 μM rosiglitazone, 500 μM IBMX 1 μM dexamethasone, 5 μg/ml insulin		0.2 μM rosiglitazone, 5 μg/ml insulin
**17.**	**10 μg/ml INS**		0.2 μM rosiglitazone, 500 μM IBMX 1 μM dexamethasone, 10 μg/ml insulin		0.2 μM rosiglitazone, 10 μg/ml insulin
	**Optimization of the maintenance medium composition**				
**18.**	**IND-4D DEX**	**4 days**	0.2 μM rosiglitazone 500 μM IBMX 1 μM dexamethasone, 10 μg/ml insulin	17 days	0.2 μM rosiglitazone, 10 μg/ml insulin, 1 μM dexamethasone
**19.**	**IND-4D IBMX**				
	**Optimization of rosiglitazone concentration**				
**20.**	**ROSI-0**	**8 days**	500 μM IBMX, 1 μM dexamethasone 10 μg/ml insulin	13 days	10 μg/ml insulin, 1 μM dexamethasone
**21.**	**ROSI-0.2**		0.2 μM rosiglitazone, 500 μM IBMX 1 μM dexamethasone, 10 μg/ml insulin		0.2 μM rosiglitazone, 10 μg/ml insulin , 1μM dexamethasone
**22.**	**ROSI-1**		1 μM rosiglitazone, 500 μM IBMX 1 μM dexamethasone, 10 μg/ml insulin		1 μM rosiglitazone, 10 μg/ml insulin, 1μM dexamethasone
**23.**	**ROSI-5**		5 μM rosiglitazone, 500 μM IBMX 1 μM dexamethasone, 10 μg/ml insulin		5 μM rosiglitazone, 10 μg/ml insulin 1μM dexamethasone
**24.**	**ROSI-10**		10 μM rosiglitazone,500 μM IBMX 1 μM dexamethasone, 10 μg/ml insulin		10 μM rosiglitazone, 10 μg/ml insulin, 1μM dexamethasone

	**Further optimization of the differentiation process with 5 µm rosiglitazone**				
**25.**	**IND-4D-R5**	4 days	5 µM rosiglitazone, 500 µM IBMX, 1 µM dexamethasone, 10 µg/ml insulin	17 days	5 µM rosiglitazone, 10ug/ml insulin
**26.**	**IND-8D-R5**	8 days		13 days	
**27.**	**IND-4D- DR5**	4 days		17 days	5 µM rosiglitazone, 10 µg/ml insulin, 1µM dexamethasone
**28.**	**IND-8D-DR5**	8 days		13 days	
**29.**	**DMEM F12**	8 days	DMEM instead DMEM F-12 5 µM rosiglitazone, 500 µM IBMX 1 µM dexamethasone, 10 µg/ml insulin	13 days	DMEM, 5%FBS, 5 µM rosiglitazone, 10 µg/ml insulin, 1 µM dexamethasone
**30.**	**10% FBS**		10% instead of 5% FBS, 5 µM rosiglitazone, 500 µM IBMX, 1 µM dexamethasone, 10 µg/ml insulin		DMEM/F-12, 10% FBS, 5 µM rosiglitazone, 10 µg/ml insulin, 1 µM dexamethasone
**31.**	**INS 20**		5 µM rosiglitazone, 500 µM IBMX 1 µM dexamethasone, 20 µg/ml insulin		DMEM/F-12, 10% FBS, 5 µM rosiglitazone, 20 µg/ml insulin, 1 µM dexamethasone
	**Optimization of the differentiation protocol with indomethacin**				
**32.**	**INDO 50**	4 days	50 µM indomethacin, 500 µM IBMX 1 µM dexamethasone, 10 µg/ml insulin	17 days	50 µM indomethacin, 10 µg/ml insulin
**33.**	**INDO 100**		100 µM indomethacin, 500 µM IBMX 1 µM dexamethasone, 10 µg/ml insulin		100 µM indomethacin, 10 µg/ml insulin
**34.**	**INDO 200**		200 µM indomethacin, 500 µM IBMX 1 µM dexamethasone, 10 µg/ml insulin		200 µM indomethacin, 10 µg/ml insulin
**35.**	**INDO 50 IBMX**		50 µM indomethacin, 500 µM IBMX 1 µM dexamethasone, 10 µg/ml insulin		100 µM indomethacin, 10 µg/ml insulin, 500 µM IBMX
**36.**	**INDO 50 DEX**		100 µM indomethacin, 500 µM IBMX 1 µM dexamethasone, 10 µg/ml insulin		100 µM indomethacin, 10 µg/ml insulin, 1µM dexamethasone
